# Ceftazidime/avibactam and aztreonam combined with an aminoglycoside combat metallo-β-lactamase-producing *Klebsiella pneumoniae*

**DOI:** 10.1128/aac.01540-25

**Published:** 2026-02-04

**Authors:** Arunkumar Karunanidhi, Nidhi Singh, Jackson V. Watkins, Nicholas M. Smith, Yanan Zang, Xindi Shan, Gilberto Espinoza, Jian Li, Yinzhi Lang, Jürgen B. Bulitta, Zackery P. Bulman

**Affiliations:** 1 Department of Pharmacy Practice, University of Illinois Chicago Retzky College of Pharmacy 14681, Chicago, Illinois, USA; 2 Department of Pharmacy Practice, School of Pharmacy and Pharmaceutical Sciences, University at Buffalo, The State University of New York 15497, Buffalo, New York, USA; 3 Department of Pharmacotherapy and Translational Research, College of Pharmacy, University of Florida 662907, Orlando, Florida, USA; 4 Department of Microbiology, Biomedicine Discovery Institute, Monash University 214149, Clayton, Victoria, Australia; Providence Portland Medical Center, Portland, Oregon, USA

**Keywords:** *Klebsiella pneumoniae*, metallo-β-lactamase, amikacin, plazomicin, aztreonam, ceftazidime/avibactam, hollow fiber infection model, combinations

## Abstract

There is an urgent need for additional therapeutic solutions to treat serious bacterial infections caused by metallo-β-lactamase (MBL)-producing *Klebsiella pneumoniae*. Aztreonam, when partnered with ceftazidime/avibactam, has *in vitro* activity against most MBL-producing *K. pneumoniae*, but clinical outcomes may be suboptimal. The purpose of this study was to determine if aminoglycosides can enhance the pharmacodynamic activity of ceftazidime/avibactam plus aztreonam against MBL-producing *K. pneumoniae*. *K. pneumoniae* isolates harboring MBL genes (*bla*_NDM_, *bla*_VIM_, and *bla*_IMP_) were evaluated in time-kill assays (*n* = 4 isolates) and the hollow fiber infection model (HFIM, *n* = 2 isolates). Clinically relevant doses of antibiotics were simulated in the HFIM, and observed antibiotic exposures represented the upper end of the expected patient plasma concentrations. Resistance was tracked during the HFIM, and confocal microscopy was used to assess cell morphology following antibiotic exposure. Both ceftazidime/avibactam + aztreonam and plazomicin were effective individually at reducing viable bacterial counts of susceptible MBL-producing *K. pneumoniae*, particularly in the HFIM. Combination regimens with aminoglycosides (amikacin or plazomicin) occasionally demonstrated synergy using traditional definitions based on viable colony counting, but they were able to consistently reduce bacterial turbidity and the emergence of filamentous cells that were observed during exposure to ceftazidime/avibactam + aztreonam. Combinations between ceftazidime/avibactam, aztreonam, and an aminoglycoside are a potentially promising therapeutic strategy to combat the rising threat posed by MBL-producing *K. pneumoniae*.

## INTRODUCTION

There has been an alarming increase in the prevalence of infections caused by metallo-β-lactamase (MBL)-producing pathogens, including *Klebsiella pneumoniae*, and few treatment options are available (1, 2). A majority of MBL-producing *K. pneumoniae* co-produce extended spectrum β-lactamases (ESBLs) or serine carbapenemase enzymes (3). This combination of β-lactamases typically renders the bacteria resistant to all available β-lactams and monobactams. However, the combination of aztreonam and avibactam maintains activity against most MBL-producing *K. pneumoniae* since the monobactam aztreonam is stable to hydrolysis by MBLs, while avibactam protects aztreonam from hydrolysis by most ESBLs and serine carbapenemases. In a panel of more than 1,000 CREs from around the world, this combination was active against 99.5% of isolates (using an MIC susceptibility cutoff of ≤4 mg/L) (4). Aztreonam/avibactam has also demonstrated clinical efficacy in patients with complicated intra-abdominal infections or pneumonia (5). Prior to the approval of aztreonam/avibactam, aztreonam combined with ceftazidime/avibactam was a preferred treatment option for MBL-producing Enterobacterales (6). Although aztreonam/avibactam is an improvement over prior treatment options for MBL-producing *K. pneumoniae*, unfavorable microbiological responses occurred in 53% of HAP-VAP patients in Phase III trials (same rate as the comparator, meropenem), and mortality rates ranged from 19% to 36% in real-world observational studies (7–9). Additionally, MBL-producing *K. pneumoniae* that co-produce AmpC genes (e.g., *bla*_CMY_) may be prone to developing aztreonam/avibactam resistance (10). In the Phase III study, bacterial persistence with an increasing MIC was reported in 6% of patients (*n* = 2/36) with HAP/VAP in the aztreonam/avibactam group (5). Aztreonam/avibactam also demonstrated poorer clinical cure rates against *K. pneumoniae* than the comparator in this study, though the sample size was small. Finally, aztreonam/avibactam has demonstrated an inability to consistently achieve >1 log_10_ CFU/mL killing of *K. pneumoniae* in preclinical murine thigh infection models, which is in contrast to its more impressive activity against *Escherichia coli* (11, 12). Taken together, these data suggest that there is a critical need to augment therapy for some patients (13).

Combining aztreonam/avibactam with another antibiotic, such as an aminoglycoside, may potentiate and prolong its clinical utility. A third antibiotic is frequently administered with ceftazidime/avibactam and aztreonam to patients with MBL-producing Enterobacterales infections (7). However, there are few data to support the selection of the third antibiotic for these patients. The addition of polymyxin B to aztreonam plus ceftazidime/avibactam has been observed to significantly reduce lung weight in a rabbit pneumonia model over 7 days, indicating a reduction in bacterial-mediated tissue injury and inflammation (14). We have previously found that aminoglycosides behave synergistically with ceftazidime/avibactam against KPC-producing *K. pneumoniae* (15, 16). Although 16S ribosomal RNA methyltransferases (16S RMTases) that confer resistance to all available aminoglycosides are not uncommon in MBL-producing Enterobacterales, aminoglycosides remain active in a high proportion of the nearly 75% of MBL producers that do not have a 16S RMTase (17). However, the interaction between aminoglycosides and aztreonam/avibactam has not been rigorously evaluated against MBL-producing *K. pneumoniae* without 16S RMTases. Here, we sought to assess the activity of aztreonam + ceftazidime/avibactam alone and in combination with aminoglycosides against MBL-producing *K. pneumoniae* using time-kill assays (TKAs) and the hollow fiber infection model (HFIM). Combinations were evaluated against MBL-producing *K. pneumoniae* that did not have a 16S RMTase, and we compared aminoglycosides with high (plazomicin) and moderate (amikacin) activity against the isolates.

## MATERIALS AND METHODS

### Bacterial isolates and antibiotic susceptibility testing

Four *K. pneumoniae* isolates that harbored MBL genes but not a 16 RMTase gene were selected for TKAs (AR#0080, AR#0135, AR#0840, and AR#0842). Two of these isolates (AR#0080 and AR#0840) were then used for HFIM studies (Table 1). MICs of aztreonam, aztreonam/avibactam (fixed avibactam concentration of 4 mg/L), aztreonam/ceftazidime/avibactam (fixed ceftazidime and avibactam concentrations of 8 and 4 mg/L, respectively), amikacin, and plazomicin were determined in triplicate using broth microdilution in CAMHB (18). Antibiotic stocks were prepared on the day of each experiment. Available susceptibility breakpoints from CLSI were used for aztreonam, amikacin, and plazomicin. The interpretations for aztreonam/avibactam (S: ≤4/4 mg/L, I: 8/4 mg/L, and R: ≥16/4 mg/L) were taken from the FDA.

**TABLE 1:** MICs for ATM, ATM//AVI, ATM/CAZ/AVI, AMK, and PLZ for MBL-producing *K. pneumoniae* isolates and their relevant antibiotic-resistance genes (β-lactamases and aminoglycoside resistance genes)

Isolate	MIC (mg/L)^*a*^					β-Lactamase genes^*b*^	Aminoglycoside resistance genes
	ATM	ATM/AVI	ATM/CAZ/AVI	AMK	PLZ		
AR#0080	≥128	0.25/4	0.5/8/4	8	0.125	***bla*_IMP-4_**, *bla*_OKP-B-21_, *bla*_OXA-1_, *bla*_SFO-1_, *bla*_TEM-1_	*aac (3)-IId, aac(6')-Ib-G, aph(3")-Ib, aph (6)-Id*
AR#0135	≥128	0.5/4	1/8/4	16	0.125	***bla*_VIM-1_**, *bla*_OXA-9_, *bla*_SHV-12_, *bla*_TEM-1A_	*aac (3)-IIa, aac(6')-Ib, aph(3')-XV*
AR#0840	128	0.06/4	0.125/8/4	8	0.5	***bla*_NDM-1_**, *bla*_CTX-M-15_, *bla*_OXA-1_, *bla*_SHV-11_, *bla*_TEM-1_	*aph(3")-Ib, aph (6)-Id*
AR#0842	≥128	0.25/4	0.25/8/4	16	0.5	***bla*_NDM-1_**, *bla*_CTX-M-15_, *bla*_OXA-232_, *bla*_OXA-9_, *bla*_SHV-1_, *bla*_TEM-1A_	*aac(6')-Ib, aadA1, aadA2*

^*a*^ AMK, amikacin; ATM, aztreonam; AVI, avibactam; CAZ, ceftazidime; PLZ, plazomicin.

^*b*^ Bold denotes metallo-β-lactamase.

### Time-kill assays (TKAs)

TKAs were conducted to initially evaluate the pharmacodynamic activity of aztreonam/ceftazidime/avibactam, amikacin, and plazomicin alone and in combinations against *K. pneumoniae* AR#0080, AR#0135, AR#0840 and AR#0842. A starting inoculum of ~10^8^ CFU/mL was used, as described previously (15, 19). Clinically relevant antibiotic concentrations were selected for testing, with the highest concentrations approximating values near the *f*C_max_ (20–22). Aztreonam/ceftazidime/avibactam was tested (30/30/4 mg/L) alone and in combinations with amikacin (25.0, 6.25, 1.56, 0.039 mg/L) or plazomicin (25, 6.25, 1.56, 0.039 mg/L). Viable colony counts were performed by obtaining samples after 0, 1, 2, 4, 6, 8, and 24 h of antibiotic exposure, serially diluting, and then plating onto MHA. Plates were incubated overnight at 37°C and then colony counts were performed. Synergy was defined as a ≥2 log_10_ CFU/mL reduction by the combination compared to the most active agent as monotherapy. Bactericidal activity was defined as a ≥3 log_10_ CFU/mL reduction in viable bacterial count compared to the initial inoculum.

### Hollow fiber infection model (HFIM)

MBL-producing *K. pneumoniae* isolates AR#0080 and AR#0840 were investigated in a 9-day HFIM as previously described with minor modifications (15, 23). Briefly, an isolate was inoculated at ~10^8^ CFU/mL in C8008 hollow fiber cartridges (FiberCell Systems Inc., Frederick, MD) at 37°C. Peristaltic pumps (Masterflex L/S; Cole-Parmer, Vernon Hills, IL) were used to provide a continuous flow of CAMHB to the central compartment throughout the experiment. Following an incubation period of ~1 h to permit log-phage growth of the *K. pneumoniae* isolates, the following regimens were initiated at 0 h: (i) amikacin (15–20 mg/kg every 24 h, 0.5-h infusion); (ii) plazomicin (15 mg/kg every 24 h, 0.5-h infusion); (iii) ceftazidime/avibactam (2.5 g every 8 h, 3-h infusion) + aztreonam (2 g every 8 h, 3-h infusion); (iv) ceftazidime/avibactam + aztreonam + amikacin; (v) ceftazidime/avibactam + aztreonam + plazomicin; and (vi) no antibiotic growth control. Human plasma pharmacokinetics for each antibiotic were mimicked in the HFIM, approximating exposures at the upper end of the normal range for average patients or closer to the median for patients with mild renal impairment (Fig. S1) (20–22, 24, 25). The antibiotics were administered to the HFIM central reservoir using automated syringe pumps (New Era Pump Systems Inc., Farmingdale, NY). Antibiotics were administered through 168 h to mimic a 7-day treatment course, after which time the remaining drug in the system was washed out by the continued flow of CAMHB. Bacterial samples were collected at 0, 1, 2, 3, 4, 6, 24, 26, 28, 30, 48, 50, 52, 54, 72, 74, 76, 78, 96, 120, 144, 168, 192, and 216 h for viable cell counting. Total bacterial counts were determined by subculturing 50 µL of serially diluted bacterial suspensions onto MHA plates.

To evaluate the potential presence of a filamentous non-replicating population following exposure to ceftazidime/avibactam and aztreonam, bacterial turbidity in the HFIM was tracked over time. Optical density measurements were obtained from samples collected from cartridges at 0, 24, 48, 72, 96, 120, 144, and 168 h. At each time point, 200 µL of bacterial culture was transferred to a 96-well clear bottom microplate (Corning Life Sciences, Tewksbury, MA), and the absorbance was read at 620 nm using a microplate reader (Cytation 5, Agilent Technologies, Inc., Santa Clara, CA).

### Emergence of resistance assay

Population analysis profiles (PAPs) were performed to detect the emergence of resistance over time. Bacterial samples collected at 0, 24, 48, 72, 96, 120, 144, and 168 h were plated on agar embedded with aztreonam/avibactam (4/4, 16/4 mg/L), amikacin (4, 16 mg/L), and plazomicin (2, 8 mg/L). Resistant colonies growing on the antibiotic-containing agar plates were quantified after 48 h of incubation.

### UPLC-MS/MS assays and modeling of pharmacokinetic data

Concentrations of the β-lactams and aminoglycosides in pharmacokinetic (PK) samples from the HFIM were quantified using an Acquity I-Class UPLC system (Waters, Milford, MA) interfaced with a Triple Quad 6500+ MS/MS system (AB Sciex, Framingham, MA) with an Acquity UPLC BEH C_18_ UPLC column (130 Å, 2.1 × 100 mm, 1.7 µm; Water). For ceftazidime, avibactam, aztreonam, and the internal standard (diclofenac), separation was carried out at 50°C with a run time of 3.5 min. The mobile phase consisted of 0.25% formic acid, 5 mM ammonium formate in water (A) and 0.1% formic acid in acetonitrile (B) at a flow rate of 0.4 mL/min. The gradient was as follows: 5% B (0–0.5 min), 5%–10% B (0.5–1.0 min), 10%–40% B (1.0–1.5 min), 40%–90% B (1.5–1.8 min), 90% B (1.8–2.8 min), 90%–5% B (2.8–2.9 min), and 5% B (2.9–3.5 min). The injection volume was 10 μL. The MS/MS system was operated in both positive (ceftazidime and aztreonam) and negative (avibactam) ion modes using the turbo spray IonDrive. Source/gas parameters were curtain gas, 30 psi; collision gas, 9 psi; ion spray voltage, +5,500 V/−4,500 V; source temperature, 550°C; and ion source gas 1 and 2, both at 45 psi. The optimized multiple reaction monitoring (MRM) transitions were *m*/*z* 436.1→313.1 for aztreonam, 547.1→468.0 for ceftazidime, and 296.0→215.1 for diclofenac, in positive mode; and *m*/*z* 264.0→96.1 for avibactam and 294.0→214.1 for diclofenac, in negative mode. Dwell time was 30 ms for all compounds.

For amikacin, plazomicin, and the internal standard (diclofenac), separation was performed using the same UPLC column at 40°C with 3.5-min run time. The mobile phase was 0.1% trifluoroacetic acid in water (A) and 0.1% formic acid in acetonitrile (B), at a flow rate of 0.4 mL/min. The gradient was 5% B (0–0.3 min), 5%–20% B (0.3–1.0 min), 20%–40% B (1.0–1.5 min), 40%–90% B (1.5–2.0 min), 90% B (2.0–2.6 min), 90%–5% B (2.6–2.8 min), and 5% B (2.8–3.5 min). The injection volume was 3 μL. The MS/MS system was operated in positive ion mode. Source/gas parameters were curtain gas, 25 psi; collision gas, 9 psi; ion spray voltage, +5,500 V; source temperature, 550°C; and ion source gas 1 and 2, both at 30 psi. MRM transitions were *m*/*z* 593.4→434.3 for plazomicin, 586.3→425.2 for amikacin, and 296.0→215.1 for diclofenac. Dwell time was 50 ms for all compounds.

Peak integration and data analysis were performed using Analyst software (AB Sciex). Samples were fivefold diluted and quantified against calibration curves generated within each analytical batch. Assay performance was validated based on established acceptance criteria (26). Assay precision/accuracy (mean %) were as follows: 21.5%/−2.9% at 0.1 mg/L (lower limit of quantification [LLOQ]), 4.0%/2.1% at 0.3 mg/L, 10.3%/−7.2% at 3 mg/L, and 9.9%/1.7% at 30 mg/L for ceftazidime; 5.6%/0.1% at 0.01 mg/L (LLOQ), 15.9%/1.1% at 0.1 mg/L, 0.8%/2.1% at 3 mg/L, and 20.4%/1.1% at 30 mg/L for avibactam; 16.4%/4.0% at 0.3 mg/L (LLOQ), 9.2%/−6.2% at 1 mg/L, 8.9%/7.4% at 3 mg/L, and 17.4%/−2% at 30 mg/L of aztreonam; 9.6%/−0.7% at 0.1 mg/L (LLOQ), 22.9%/0.3% at 0.3 mg/L, 19.4%/−7.3% at 10 mg/L, and 9.5%/3.4% at 30 mg/L of amikacin; 6.1%/6.3% at 0.01 mg/L (LLOQ), 8.9%/−11.6% at 0.1 mg/L, 20.1%/3.3% at 3 mg/L, and 8.2%/−1.8% at 30 mg/L of plazomicin.

To calculate the observed clearance and volume of distribution of the HFIM, the observed antibiotic concentrations measured by UPLC-MS/MS from the central reservoir for all five antibiotics were fit to a one-compartment model using Monolix (Simulations Plus, Inc.). In addition, 1,000 person Monte Carlo simulations (MCSs) were performed to show the population exposures of each antibiotic relative to the observed concentrations, based on previously published population pharmacokinetic studies (21, 27–29). To provide clinical context to the HFIM PK, simulations were conducted assuming creatinine clearance of 60 and 100 mL/min. MCSs were performed using RxODE and graphed using GGPlot2 using R version 4.4.1 (30).

### Confocal microscopy

To evaluate cell morphology changes for MBL-producing *K. pneumoniae* isolates following antibiotic exposure, a modified TKA followed by laser-scanning confocal microscopy was performed. Isolates AR#0080 and AR#0842 were suspended in CAMHB to obtain cultures containing ~10^7^ CFU/mL. Bacteria were exposed to aztreonam-avibactam alone (4x and 8x MIC) and in combination with amikacin (4x MIC) or plazomicin (4x MIC) for 3 h at 37°C in a shaking incubator. Untreated bacteria were included as a control. Approximately 20–30 µL of bacterial sample from each tube was collected at 3 h and then transferred to an individual 35 mm petri dish with coverslip (Mattek Corporation). Finally, cells were imaged by confocal microscopy (Zeiss LSM 710 confocal microscope) at ×63 magnification. Composite image files were loaded into Fiji (ImageJ, National Institute of Health). Brightness and contrast settings were adjusted to constrain signal and eliminate noise.

## RESULTS

### Antibiotic susceptibility testing

All isolates were resistant to aztreonam (≥128 mg/L) but susceptible to aztreonam/avibactam (≤0.5/4 mg/L) (Table 1). Each of the isolates was susceptible to plazomicin but nonsusceptible to amikacin. AR#0135 and AR#0842 were amikacin-resistant, while AR#0080 and AR#0840 displayed intermediate resistance.

### TKAs

**Fig 1:**
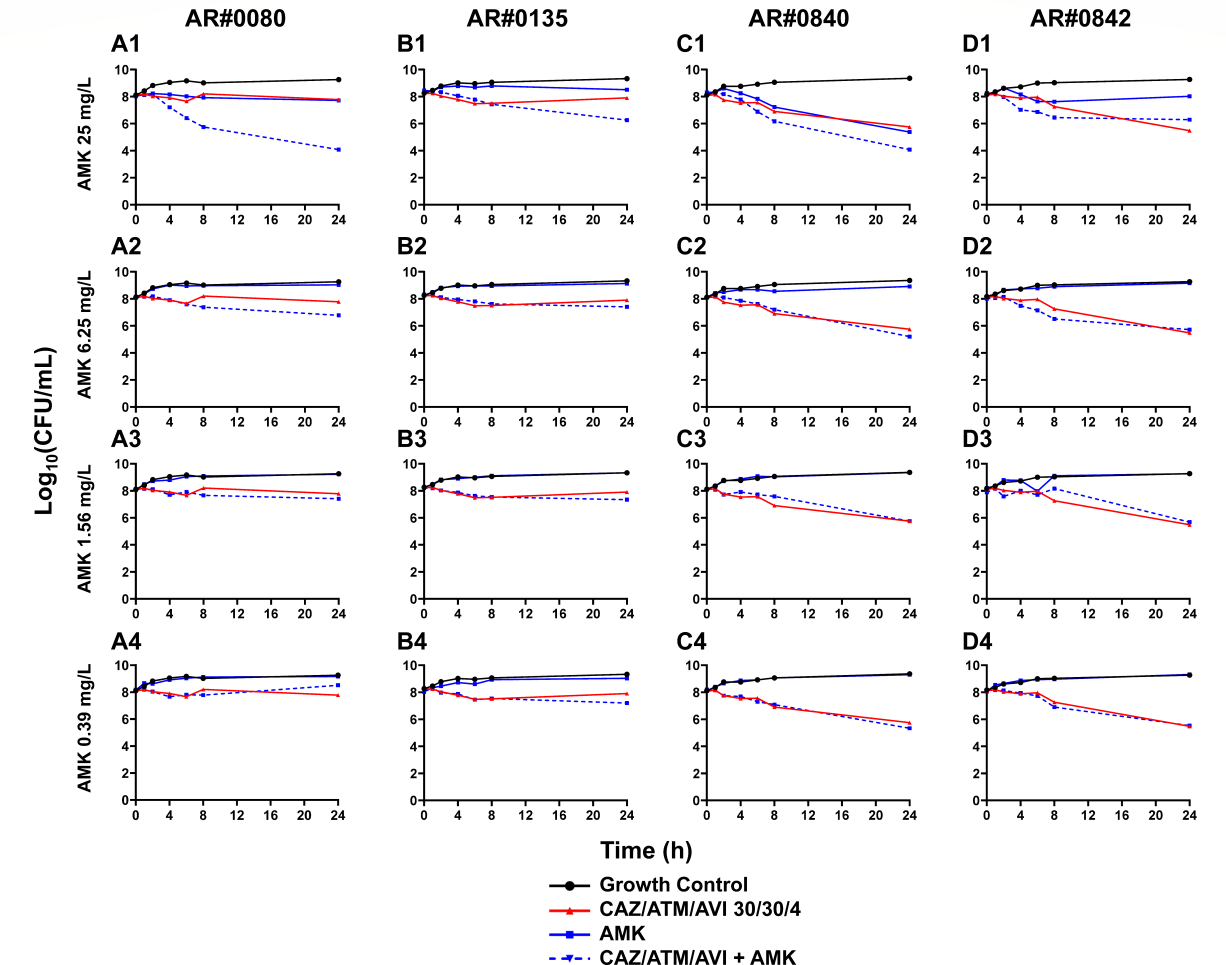
Activity of amikacin (AMK) combined with aztreonam (ATM)/ceftazidime (CAZ)/avibactam (AVI) against MBL-Kp isolates AR#0080 (**A1– A4**), AR#0135 (**B1–B4**), AR#0840 (**C1–C4**), and AR#0842 (**D1–D4**) in time-kill assays over 24 h. For each isolate, the growth control and monotherapy lines are duplicated in multiple panels to enable easier comparisons with combinations.

**Fig 2:**
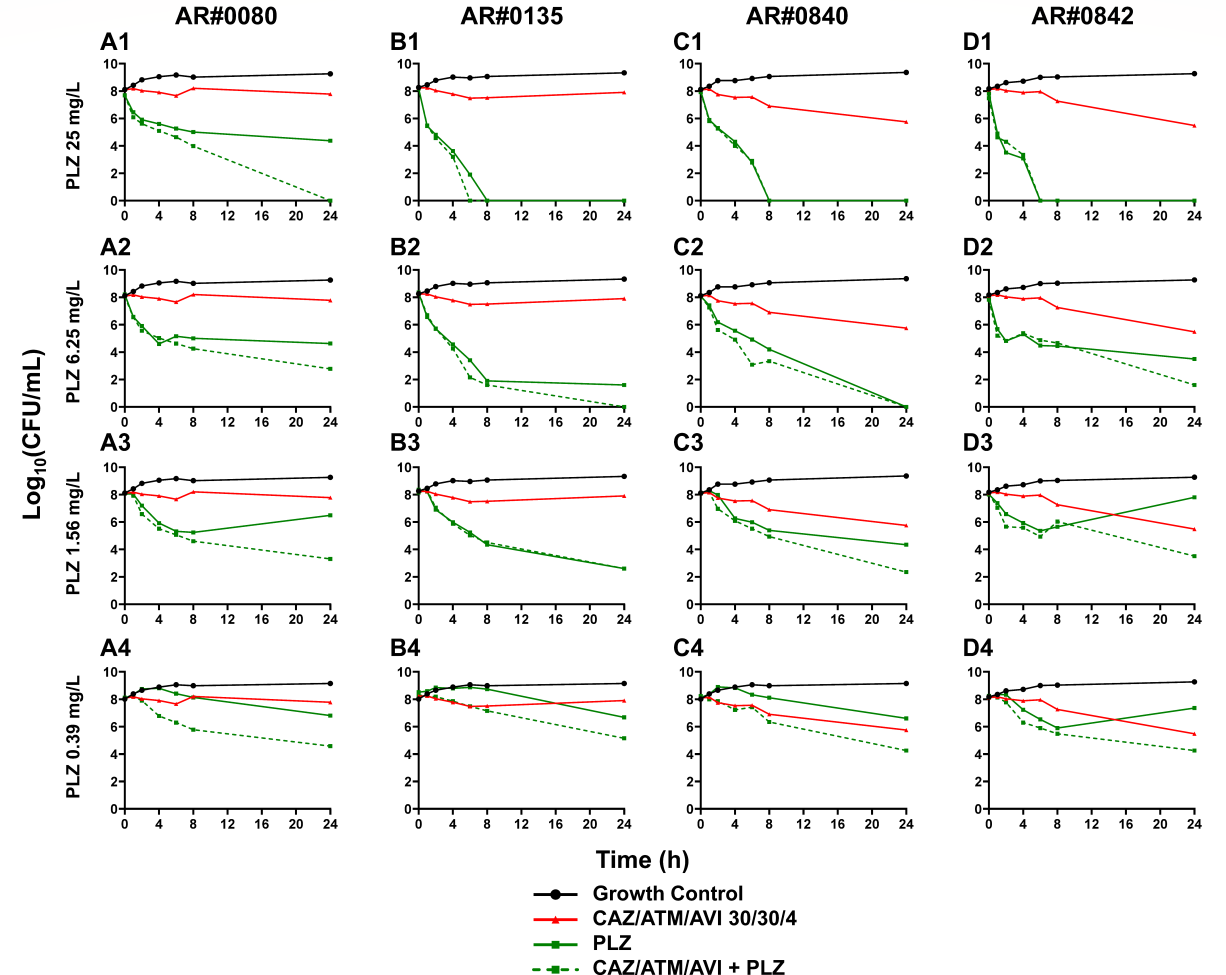
Activity of plazomicin (PLZ) combined with aztreonam (ATM)/ceftazidime (CAZ)/avibactam (AVI) against MBL-Kp isolates AR#0080 (**A1–A4**), AR#0135 (**B1–B4**), AR#0840 (**C1–C4**), and AR#0842 (**D1–D4**) in time-kill assays over 24 h. For each isolate, the growth control and monotherapy lines are duplicated in multiple panels to enable easier comparisons with combinations.

**Fig 3:**
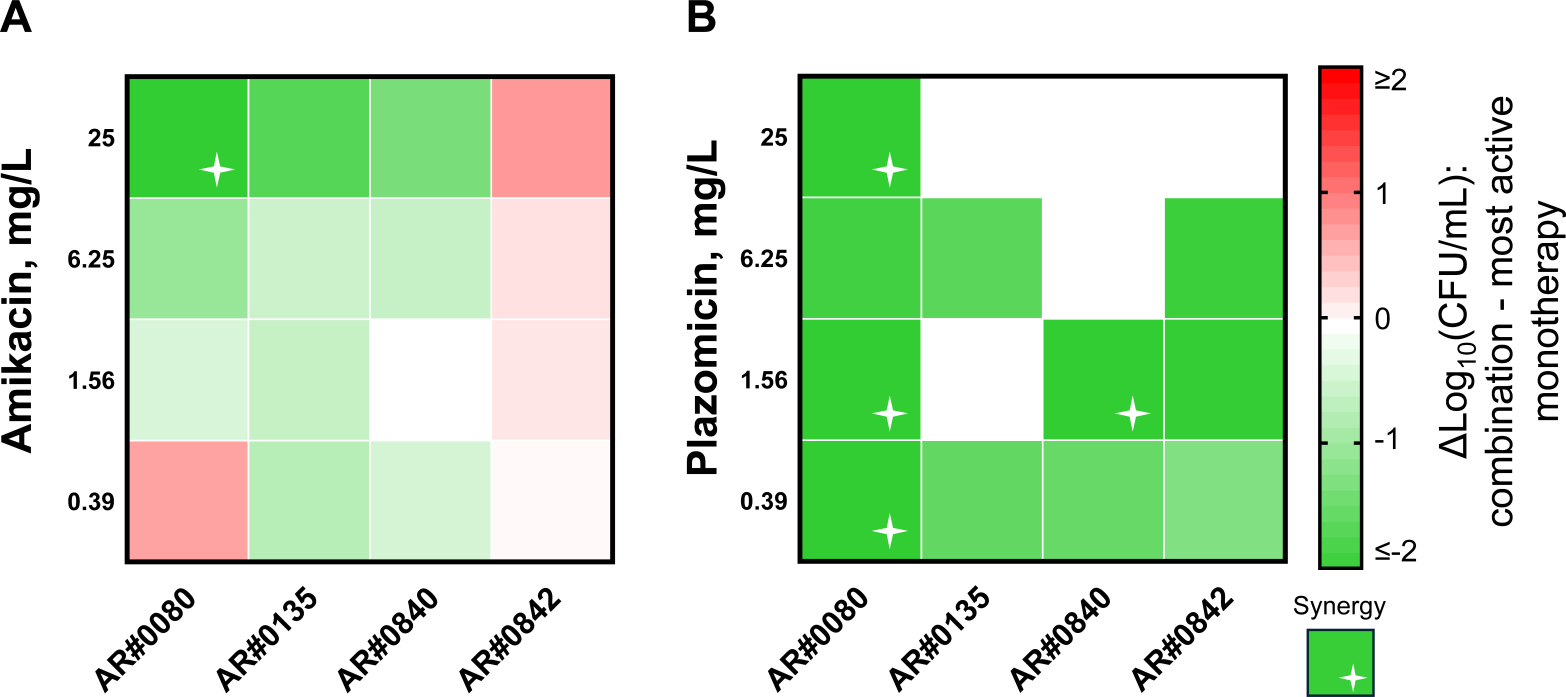
Heat map depicting interactions for combinations between aztreonam/ceftazidime/avibactam and amikacin (**A**) or plazomicin (**B**) against MBL-Kp isolates at 24 h in time-kill assays. The colors correspond to the log_10_ CFU/mL reductions by the combinations compared to the most active monotherapy, where boxes shaded green mean that the combination was more active than either agent alone, and boxes shaded in red mean that the combination was less active than at least one agent alone. Combinations that were synergistic are represented by green boxes with a symbol.

In TKAs at an inoculum of ~10^8^ CFU/mL, aztreonam/ceftazidime/avibactam at a concentration of 30/30/4 mg/L was not bactericidal against any isolate (Fig. 1 and 2) and displayed a 1.40 log_10_ CFU/mL mean bacterial killing at 24 h. Amikacin at concentrations below the MIC exhibited minimal activity, whereas 25 mg/L caused up to a 2.92 log_10_ CFU/mL reduction (Fig. 1A1 through D1). Amikacin had a minimal impact on the activity of aztreonam/ceftazidime/avibactam when amikacin concentrations were ≤6.25 mg/L (below the MIC). Amikacin at 25 mg/L increased the activity of aztreonam/ceftazidime/avibactam by ~0.5 to 1.0 log_10_ CFU/mL within the first 8 h. The combination of aztreonam/ceftazidime/avibactam with amikacin was synergistic against one isolate, AR#0080 (amikacin at 25 mg/L), at 24 h (Fig. 3A).

Against all four MBL-harboring isolates, plazomicin (25 and 6.25 mg/L) was bactericidal (Fig. 2A1 through D1 and A2 through D2). Plazomicin 25 mg/L combined with aztreonam/ceftazidime/avibactam eradicated AR#0135, AR#0840, and AR#0842 within 8 h (Fig. 2B1 through D1). Plazomicin (1.56 mg/L) was bactericidal against AR#0135 with a 5.75 log_10_ CFU/mL reduction at 24 h (Fig. 2B3). The combination of aztreonam/ceftazidime/avibactam with plazomicin met the threshold for synergy against AR#0080 (plazomicin at 25.0, 1.56, and 0.39 mg/L) and AR#0840 (plazomicin at 1.56 mg/L) at 24 h (Fig. 3B).

### HFIM

Modeling the observed antibiotic concentrations detected by LC-MS/MS in the central reservoir of the HFIM revealed a clearance of 0.026 L/h and a Vd of 0.230 L. The observed half-life of the model (6.1 h) and observed *f*C_max,ss_ values following doses of ceftazidime (90.5 mg/L), avibactam (22.5 mg/L), aztreonam (60.0 mg/L), amikacin (29.9 mg/L), and plazomicin (28.5 mg/L) put the antibiotic exposures at the upper end of the normal range for average patients or closer to the median for patients with mild renal impairment (Fig. S1). Antibiotic concentrations in the HFIM cartridges were generally consistent with those in the central reservoir (Fig. S1 and S2). Aztreonam and avibactam concentrations were equivalent in the central reservoir and cartridge throughout the pharmacokinetic studies. Ceftazidime, amikacin, and plazomicin had a detectable distribution phase, where lower concentrations in the cartridge were observed during the first 1 h of dosing.

**Fig 4:**
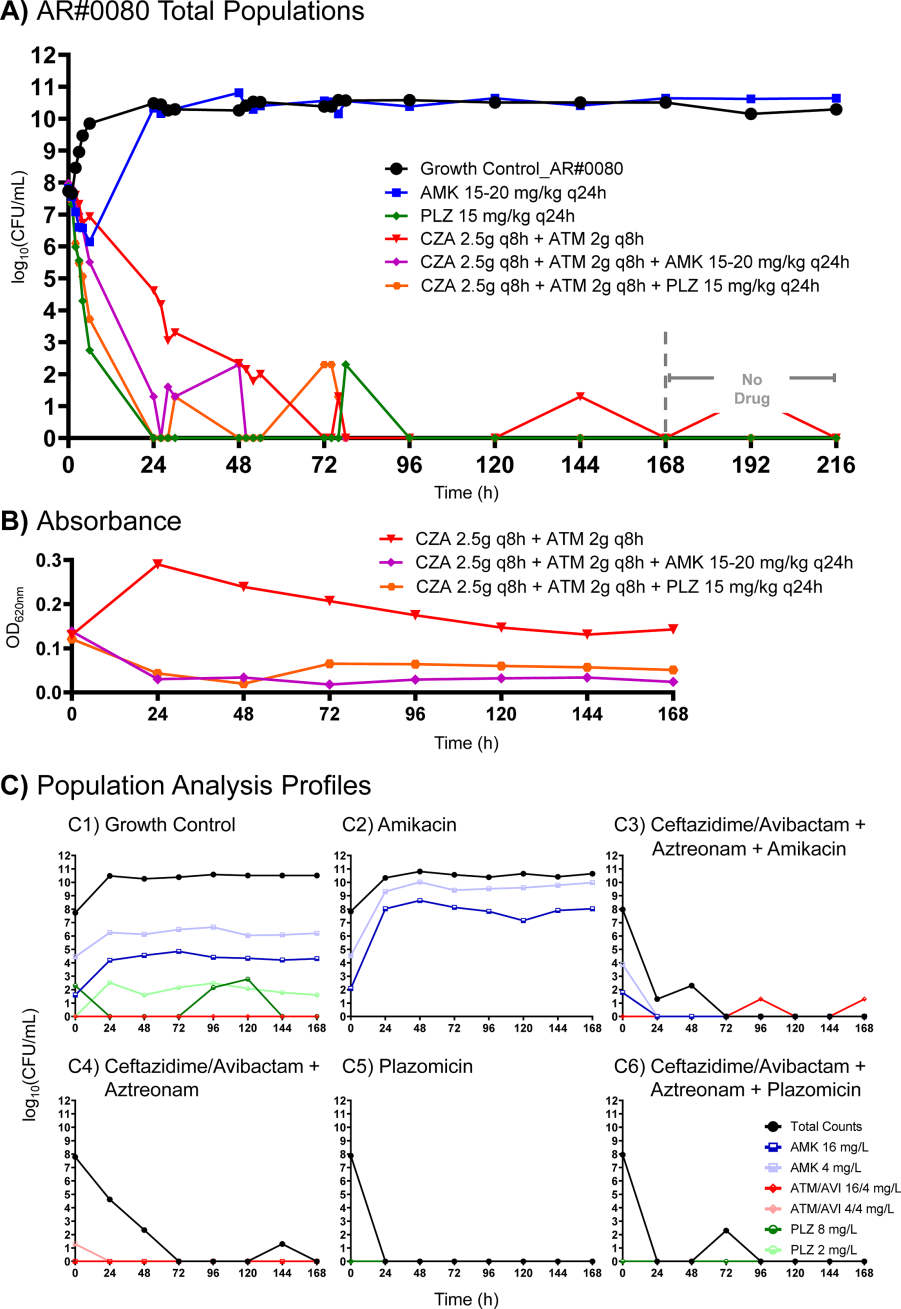
Bacterial viability and resistance time course of AR#0080 in the 9-day HFIM following exposure to various monotherapy and combination therapy. Drug administration was discontinued after the 168-h time point (dashed line), and the experiment was continued for an additional 48 h to assess population viability upon return of favorable growth conditions. Bacteria were profiled over time as total populations grown on drug-free agar (**A**), absorbance measured at OD_620nm_ (**B**), and population analysis profiles grown on drug-embedded agar (**C**).

For AR#0080, aztreonam + ceftazidime/avibactam caused 3.17 log_10_ CFU/mL reductions at 24 h (Fig. 4A). This antibiotic regimen drove bacterial counts to become undetectable by 72 h, and counts remained <10^2^ CFU/mL through the rest of the experiment. Amikacin monotherapy caused a 1.68 log_10_ CFU/mL reduction at 6 h but regrew to 10.64 log_10_ CFU/mL by 168 h. In contrast, plazomicin monotherapy demonstrated a 5.13 log_10_ reduction in CFU/mL at 6 h, which was 4.18 log_10_ CFU/mL greater than that of aztreonam + ceftazidime/avibactam at this time. Bacterial counts were undetectable through the rest of the experiment aside from a single time point (78 h). Aztreonam + ceftazidime/avibactam in combination with amikacin caused bacterial reductions of 2.47 and 6.68 log_10_ CFU/mL at 6 and 24 h, respectively. This combination was 0.63 and 3.32 log_10_ CFU/mL more active at 6 and 24 h than the most active monotherapy at each time point. The combination of aztreonam + ceftazidime/avibactam with plazomicin achieved killing similar to plazomicin monotherapy and slightly better than the combination of aztreonam + ceftazidime/avibactam with amikacin. Aztreonam + ceftazidime/avibactam with plazomicin caused bacterial reductions of 4.23 and 7.95 log_10_ CFU/mL at 6 and 24 h, respectively.

**Fig 5:**
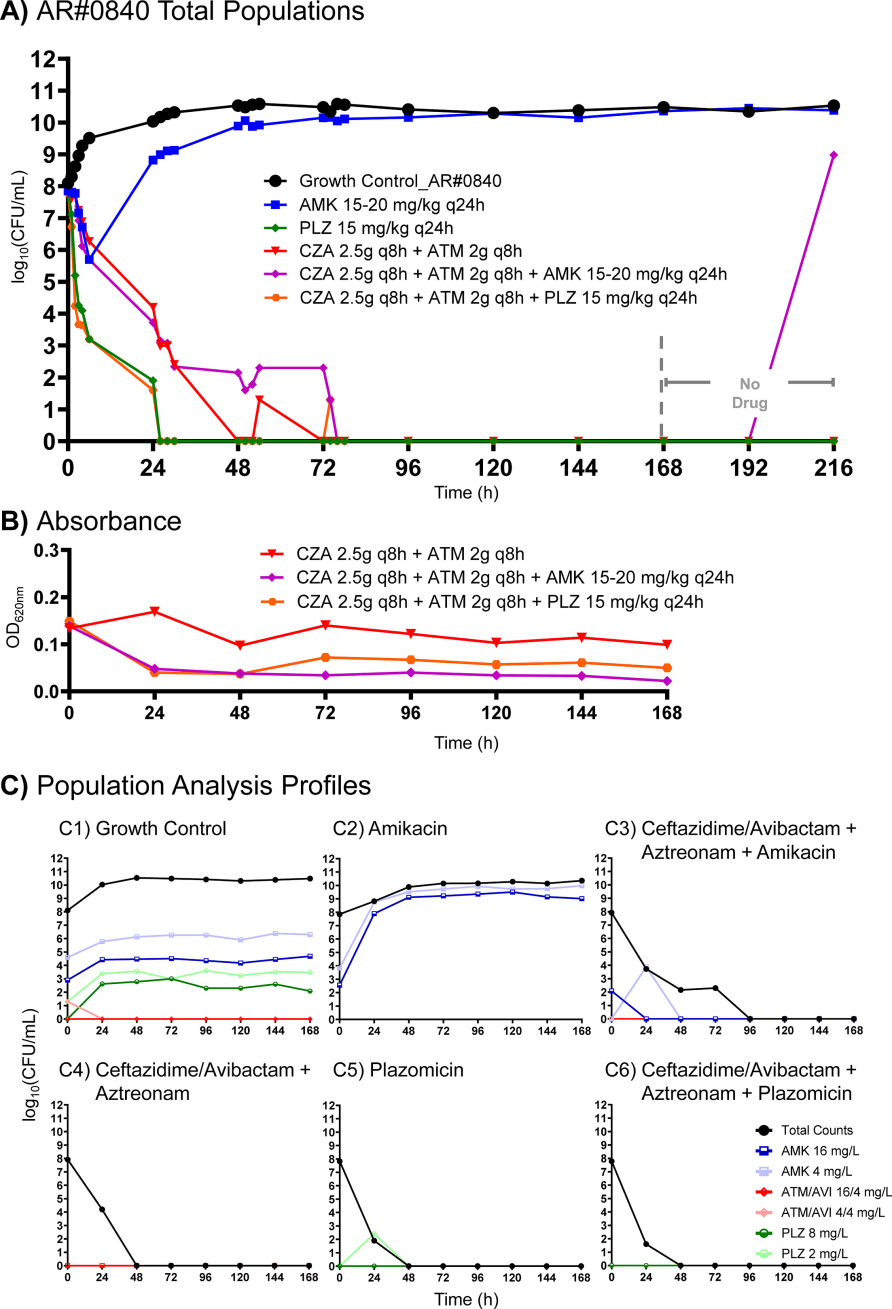
Bacterial viability and resistance time course of AR#0840 in the 9-day HFIM following exposure to various monotherapy and combination therapy. Therapeutic regimens were simulated over 168 h. Drug administration was discontinued after the 168-h time point (dashed line), and the experiment was continued for an additional 48 h to assess population viability upon return of favorable growth conditions. Bacteria were profiled over time as total populations grown on drug-free agar (**A**), absorbance measured at OD620nm (**B**), and population analysis profiles grown on drug-embedded agar (**C**).

Against AR#0840 in the HFIM, aztreonam + ceftazidime/avibactam caused bacterial reductions of 3.72-log_10_ CFU/mL at 24 h (Fig. 5A). Bacterial counts were undetectable from 72 h through the end of the experiment during exposure to aztreonam + ceftazidime/avibactam. Similar to AR#0080, amikacin monotherapy caused a 2.15 log_10_ CFU/mL reduction at 6 h against AR#0840 but regrew thereafter. Meanwhile, plazomicin monotherapy caused 4.62 and 5.92 log_10_ CFU/mL reductions at 6 and 24 h, respectively, and no growth was observed after that time. Adding amikacin to aztreonam + ceftazidime/avibactam failed to appreciably reduce viable bacterial counts beyond what was observed for aztreonam + ceftazidime/avibactam. The killing pattern of aztreonam + ceftazidime/avibactam + plazomicin was remarkably similar to that of plazomicin alone, causing bacterial reduction of 4.58 and 6.18 log_10_ CFU/mL at 6 and 24 h, respectively.

Notable differences pertaining to the absorbance/turbidity were detected in the HFIM for both AR#0080 and AR#0840 (Fig. 4B and 5B). When the isolates were exposed to ceftazidime/avibactam and aztreonam, turbidity increased (AR#0080) or remained relatively constant (AR#0840) over time. In contrast, when amikacin or plazomicin was added to ceftazidime/avibactam and aztreonam, the turbidity decreased by 24 h and remained lower than ceftazidime/avibactam and aztreonam through 168 h.

### Emergence of resistance assay

Regrowth after exposure to amikacin monotherapy corresponded to amplification of aminoglycoside-resistant subpopulations for both AR#0080 and AR#0840, even on plates containing the highest concentration of amikacin (16 mg/L) (Fig. 4C2 and 5C2). In contrast, we observed suppression of plazomicin-resistant subpopulations after 24 h, which corresponded to the enhanced killing we observed following treatment with this aminoglycoside (Fig. 4C5 and 5C5). No resistant colonies appeared on aztreonam/avibactam PAPs for any treatment regimen apart from growth detected at two time points on aztreonam/avibactam plates (16/4 mg/L) for AR#0080 when it was exposed to ceftazidime/avibactam + aztreonam + amikacin (Fig. 4C3). This was also the lone regimen that regrew following antibiotic discontinuation.

### Confocal microscopy

**Fig 6:**
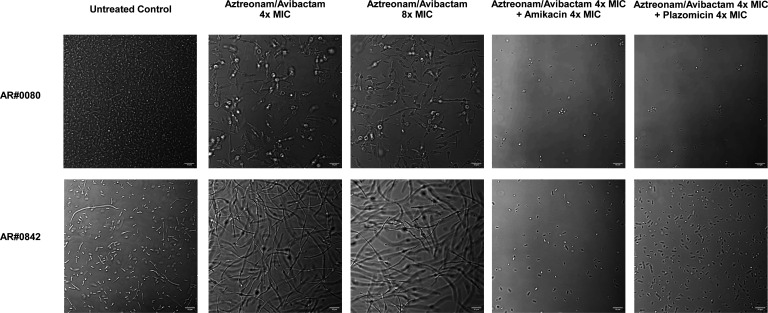
Images of two MBL-producing *K. pneumoniae* following exposure to aztreonam/avibactam alone or in combination with an aminoglycoside using confocal microscopy. Images were taken 3 h after antibiotic exposure was initiated.

MBL-producing *K. pneumoniae* exposed to aztreonam/avibactam were elongated and filamentous, which was not observed in bacteria exposed to aminoglycosides or in the untreated control (Fig. 6). Elongated cells generally ranged from 15 to 20 µm in length, compared to the 1–2 µm observed in our controls. In some rare instances, filaments measured as long as 25–30 µm. Remarkably, adding an aminoglycoside to aztreonam/avibactam was able to prevent the formation of the filamentous cells.

## DISCUSSION

Aztreonam/avibactam or ceftazidime/avibactam combined with aztreonam are considered to be standard of care therapeutic options for MBL-producing Enterobacterales, although clinical outcomes are not yet fully optimized, and some opportunity for improvement remains (31). Thus, we sought to determine if aminoglycosides may enhance the activity of aztreonam/ceftazidime/avibactam using TKAs and the HFIM. Both aztreonam+ ceftazidime/avibactam and plazomicin were highly active against MBL-producing *K. pneumoniae* in the HFIM, and combination therapies were only occasionally synergistic in TKAs or HFIM when applying standard definitions based on viable colony counts (i.e., CFU/mL). However, in the absence of an aminoglycoside, aztreonam + ceftazidime/avibactam HFIM cartridges remained noticeably turbid despite limited viable counts on agar plates, suggesting the presence of a non-replicating population, as has been previously observed for this therapy (23, 32, 33). In contrast, turbidity was not detected when an aminoglycoside was added. Confocal microscopy confirmed that the addition of an aminoglycoside was able to minimize the formation of a non-replicating filamentous population. To our knowledge, we are the first to investigate combinations with aminoglycosides and aztreonam + ceftazidime/avibactam against MBL-producing *K. pneumoniae* in a dynamic pharmacokinetic/pharmacodynamic model and the first to report the activity for any dosing regimen of plazomicin in the HFIM.

Aminoglycosides are generally considered to be rapidly bactericidal, and resistance in Enterobacterales is primarily caused by aminoglycoside-modifying enzymes (AMEs), which are commonly co-harbored by MBL producers (17, 34). Plazomicin is a potent aminoglycoside with promising activity against multidrug-resistant *K. pneumoniae* that produces β-lactamases and carbapenemases, including MBLs. Unlike amikacin and other available aminoglycosides, plazomicin is known to evade most clinically relevant AMEs in *K. pneumoniae* and has been found to be active in more than 71%–90% of the VIM-producing and 66%–100% of the NDM-producing *K. pneumoniae* (17, 35). In contrast, only about a third of these isolates are susceptible to amikacin or gentamicin. The aminoglycoside susceptibility patterns in MBL-producing *K. pneumoniae* are contingent on the local prevalence of 16S RMTases, which can vary (36, 37). We excluded 16S RMTases from the present study, but each of the isolates encoded an allele of *aac(6ʹ)-lb*, which encodes an acetyltransferase that can acetylate amikacin and was likely responsible for its elevated MICs compared to plazomicin (19, 38). This enabled us to compare two aminoglycosides with varying activity. As monotherapy, amikacin displayed modest initial activity, which was followed by the emergence of resistance and regrowth in the HFIM, whereas plazomicin was remarkably active as monotherapy. Due to differences in aminoglycoside MIC, the relative exposures of plazomicin were 16–64× more than amikacin for the strains tested in the HFIM. Thus, it is not surprising that amikacin monotherapy failed to provide sustained killing since exposures were suboptimal against these isolates (*f*C_max_/MIC = 3.8 and *f*AUC/MIC = 14.7) (39). Plazomicin exposures during a simulated 15 mg/kg q24h dose delivered excellent activity as monotherapy in the HFIM, and exposures were in excess of the PK/PD targets (*f*C_max_/MIC >60 and *f*AUC/MIC >235). Our findings are consistent with a previous study showing plazomicin exposures with an *f*AUC/MIC of >132 suppress the emergence of resistance in *K. pneumoniae* (40).

Aztreonam/avibactam displays potent *in vitro* activity against a broad array of MBL-producing *K. pneumoniae* (12, 41). Utilizing a clinically relevant concentration of 30/30/4 mg/L for aztreonam/ceftazidime/avibactam resulted in moderate and gradual killing of all four MBL-producing *K. pneumoniae* isolates in TKAs, with a mean log_10_CFU/mL reduction of 1.40. Previous studies have found that this β-lactam/β-lactamase inhibitor combination is capable of causing ~3 to 4 log_10_CFU/mL reductions against MBL-producing Enterobacterales, though they used a starting inoculum that was 100× lower than in our study (i.e., 10^6^ CFU/mL), while we used 10^8^ CFU/mL to simulate a more severe infection (42, 43). In contrast to the limited killing observed in the TKAs, ceftazidime/avibactam + aztreonam resulted in ~8 log_10_CFU/mL reduction within 48–72 h in the HFIM. An inoculum of 10^8^ CFU/mL is much closer to the bacterial carrying capacity of the TKA (~10^9^ CFU/mL) than for the HFIM (~10^10^ CFU/mL). As a result, the growth rate of each isolate was initially much faster in the HFIM, which likely contributes to the enhanced kill rate achieved by aztreonam/ceftazidime/avibactam in this model . The rate of bacterial killing by β-lactams is correlated in a linear fashion with the growth rate of the isolate (44). Post hoc TKAs performed at lower starting inocula with AR#0080 and AR#0840 revealed that there was a linear correlation between the rate of killing by aztreonam/ceftazidime/avibactam and the rate of growth, lending support to this hypothesis (Fig. S3). The reduced growth rate and consequent attenuated killing observed at higher inocula may, in part, explain the inoculum effect for this antibiotic. An inoculum effect has also been observed *in vivo* for aztreonam/avibactam against *E. coli* (33, 45). Smith et al. have previously shown that ceftazidime/avibactam + aztreonam against MBL-producing *K. pneumoniae* in the same experimental model killed more gradually and maxed out at a reduction between ~4.5 and 7.5 log_10_CFU/mL (23). This difference may be due to the different antibiotic exposure profiles tested in the present study. Here, we tested a 3-h infusion duration (versus 2-h infusions in some previous studies) and mimicked pharmacokinetics of patients with lower antibiotic clearance (e.g., impaired renal function), suggesting that prolonged or continuous infusion approaches may have pharmacodynamic advantages for this combination.

Aminoglycosides have frequently been observed to exert a synergistic effect with β-lactams. For example, plazomicin was found to interact synergistically with piperacillin/tazobactam and ceftazidime against an array of Enterobacterales strains (46). However, in the very few studies that have evaluated combinations between aztreonam and aminoglycosides in *K. pneumoniae*, checkerboard testing was used, and synergy was not frequently observed (47, 48). This is consistent with what we found in the present study, where there was a somewhat limited degree of synergy observed when aminoglycosides were combined with aztreonam/ceftazidime/avibactam. In the present study, combinations of aztreonam/ceftazidime/avibactam with plazomicin were often more active than either agent alone in TKAs, though this only occasionally met the defined threshold for synergy. Combinations with amikacin were more active than either monotherapy in the HFIM against 1 of 2 tested isolates (AR#0080). In agreement with this finding, amikacin produced the greatest enhancement of aztreonam/ceftazidime/avibactam activity against AR#0080 among strains tested in TKAs. In contrast, amikacin did less to potentiate AR#0840 in both TKAs and in the HFIM. Although the magnitude of killing somewhat differed between TKAs and HFIM for individual antibiotics (e.g., aztreonam/ceftazidime/avibactam killed less in TKAs), the relative activity of the combinations was generally consistent. However, the remarkable activity of plazomicin monotherapy in the HFIM made it difficult to assess its interaction with aztreonam/ceftazidime/avibactam. Combinations between plazomicin and aztreonam/ceftazidime/avibactam may prove more valuable against MBL-producing *K. pneumoniae* that have higher MICs to either of these antibiotics.

Although combination regimens failed to consistently demonstrate synergy using traditional definitions based on viable colony counting in our study, differences were observed compared to monotherapy. Specifically, turbidity in the HFIM cartridges following treatment with aztreonam/avibactam or ceftazidime/avibactam + aztreonam was observed in spite of viable bacterial counts that were undetectable. Turbidity was likely due to the presence of filamentous *K. pneumoniae* that were unable to divide due to the inhibition of penicillin-binding protein 3 (PBP3) by aztreonam, which our group (33) and others (23, 32) have observed previously. PBP3 is essential for cell septation as it is responsible for catalyzing the cross-linking of peptidoglycan chains in the cell’s divisome. Filamentous cells have been shown to release significantly more endotoxins than the spheroplasts that typically form following PBP2 inhibition (49). Interestingly, when an aminoglycoside (amikacin or plazomicin) was added to the aztreonam-based treatment regimens, turbidity was reduced to levels similar to the blank broth. This suggests that the aminoglycosides were able to continue killing the filamentous and non-replicating aztreonam-exposed bacteria, which may be a result of continued cell translation despite not being able to actively divide (50) and may have implications for the treatment of biofilms (51).

There are several notable limitations to the current project. First, replicate HFIM experiments were not performed. Thus, pharmacodynamic differences between regimens, particularly those that are relatively small, should be interpreted with appropriate caution. Second, none of the tested isolates contained a 16S RMTase, which confers high-level resistance to each available aminoglycoside and can be co-harbored by MBL-producing *K. pneumoniae*. Thus, further studies are required to determine if there is any benefit of adding an aminoglycoside to aztreonam/ceftazidime/avibactam when treating isolates with these resistance determinants. Lastly, aztreonam concentrations were occasionally above those predicted for patients with impaired renal function. However, reduced aztreonam clearance during co-administration with ceftazidime/avibactam has been previously observed in Phase I studies of this combination, suggesting that the concentrations may plausibly reflect those encountered in patients (25).

Optimizing treatment approaches for infections caused by MBL-producing *K. pneumoniae* is of critical importance due to their increasing prevalence globally. Aztreonam/avibactam may have suboptimal treatment outcomes when used alone against MBL-producing *K. pneumoniae*, highlighting the potential importance of using it in combination. Herein, we show that ceftazidime/avibactam + aztreonam and plazomicin are each effective individually at reducing viable bacterial counts of susceptible MBL-producing *K. pneumoniae*. It is important to note that the antibiotic doses simulated herein mimicked patients toward the upper end of the normal pharmacokinetic profiles. Thus, in patients with faster drug clearance, altered dosing strategies may be necessary to achieve comparable pharmacodynamic activity. Nevertheless, a population of filamentous cells appeared after treatment with aztreonam + ceftazidime/avibactam alone, which was prevented with the addition of aminoglycosides. Combinations between ceftazidime/avibactam, aztreonam, and an aminoglycoside are a promising therapeutic strategy to combat the rising threat posed by MBL-producing *K. pneumoniae*.

## SUPPLEMENTAL MATERIAL

Supplemental material aac.01540-25-s0001.docx Fig. S1 to S3.

ASM does not own the copyrights to Supplemental Material that may be linked to, or accessed through, an article. The authors have granted ASM a non-exclusive, world-wide license to publish the Supplemental Material files. Please contact the corresponding author directly for reuse.
