# Supplementary material for: Ceftazidime/avibactam and aztreonam combined with an aminoglycoside combat metallo-β-lactamase-producing Klebsiella pneumoniae
Source: Antimicrob Agents Chemother. 2026 Feb 4;70(3):e01540-25. doi: 10.1128/aac.01540-25 (PMC12959111; doi:10.1128/aac.01540-25)
Supplement: Supplemental material — Fig. S1 to S3. [file aac.01540-25-s0001.docx]

**SUPPLEMENTAL INFORMATION**

**Figure S1.** Observed antibiotic concentrations determined by LC-MS/MS for samples from the central reservoir (diamond symbol) and cartridge (circle symbol) in the hollow fiber infection model. Simulated patient exposures for each antibiotic were determined by performing Mote Carlo simulations anchored around patients with normal renal function (100 ml/min; teal) or impaired renal function (60/mL/min; pink). Clinically relevant doses of aztreonam (2000 and 2666 mg, q8h), ceftazidime (2000 mg, q8h), avibactam (500 mg, q8h), amikacin (15 mg/kg, q24h), and plazomicin (15 mg/kg, q24h) were simulated.

**Figure S2.** Forest plot of the cartridge-to-reservoir concentration ratio in the hollow fiber infection model. Geometric mean ratios (GMR) for observed concentrations of antibiotics in the cartridge compared to the central reservoirs are depicted as circles with 90% CI. The GMR was calculated by taking the natural log of the ratio of concentrations in the cartridge to concentrations in the central reservoir, averaging, and then back transforming. Values <1 represent instances where concentrations in the cartridge were lower than those in the central reservoir. The pre-specified equivalence band of 0.80-1.25 is shaded and equivalence is concluded when the entire 90% CI falls within the bands. GMRs were calculated for the entire pharmacokinetic sampling window (48 hours) and after the initial distribution phase (1 hour). All drugs except plazomicin achieved equivalence for at least the 1.5-48 h sample window.

**Figure S3.** Time-kill assays with aztreonam/ceftazidime/avibactam against AR#0080 (**A1**) and AR#0840 (**A2**) at 4 different starting inocula. There was a linear correlation between the initial growth rate of the bacteria in the growth control group and the initial kill rate for aztreonam/ceftazidime/avibactam (**B1 and B2**). Initial growth rates and kill rates were quantified over the first 4 hours by dividing the change in log_10_ CFU/mL by 4 hours. Initial growth and kill rates were also calculated the same way using data from the HFIM and overlaid on the graph for comparison (square shape).
